# Supplementary material for: High occurrence of transportation and logistics occupations among vascular dementia patients: an observational study
Source: Alzheimers Res Ther. 2019 Dec 27;11:112. doi: 10.1186/s13195-019-0570-4 (PMC6933928; doi:10.1186/s13195-019-0570-4)
Supplement: Supplementary file 1 — Additional file 1. Overview of occupational classes and examples. [file 13195_2019_570_MOESM1_ESM.docx]

**Overview of occupational classes and examples**

**1. Pedagogical**

- Teachers in primary/secondary education

- Sports instructors

- Kindergarten teachers

- Teaching assistants

**2. Creative/Linguistic**

- Authors/journalists

- Librarians

- Visual/performing artists

- Interior designers

**3. Commercial**

- Advisors marketing/public relations

- Representatives

- Sales persons

- Call center employees

**4. Business/Administrative**

- Accountants

- Economists

- Policy advisors

- Administrative assistants

**5. Management**

- Managing directors

- School directors

- Healthcare facility managers

- Wholesale managers

**6. Government/Safety/Law**

- Government officials

- Lawyers

- Police officers

- Military personnel

**7. Technical**

- Engineers

- Construction workers

- Bakers/butchers- Electricians

- Assembly workers

**8. ICT**

- Software developers

- Database specialists

- Radio/television technicians

**9. Agricultural**

- Horticulturists

- Farmers

- Breeders/growers

**10. Health Care/Welfare**

- Doctors

- Nurses

- Psychologists

- Social workers

- Medical laboratory technicians

**11. Service**

- Cooks

- Hairdressers

- House keepers/janitors

- Tour guides

**12. Transportation/Logistics**

**-** Truck/taxi/bus drivers

- Train operators

- Deck officers

- Warehouse workers
