# Supplementary material for: High occurrence of transportation and logistics occupations among vascular dementia patients: an observational study
Source: Alzheimers Res Ther. 2019 Dec 27;11:112. doi: 10.1186/s13195-019-0570-4 (PMC6933928; doi:10.1186/s13195-019-0570-4)
Supplement: Supplementary file 2 — Additional file 2: Table S1. Clinical criteria for different dementia types between 2000-2017. [file 13195_2019_570_MOESM2_ESM.pdf]

Table S1. Clinical criteria for different dementia types between 2000-2017

**AD**	McKhann et al. (NINCDS-ADRDA) [24]; McKhann et al. (NIA-AA) [25]
**FTD**	Neary et al. [26]; Rascovsky et al. (behavioral variant) [27] and Gorno-Tempini et al. (primary progressive aphasia) [28]
**VaD**	Román et al. (NINDS-AIREN) [29]
**DLB**	McKeith et al. [30]; McKeith et al [31]
**PSP**	Litvan et al. (NINDS-SPSP) [32]
**CBD**	Boeve et al. [33]; Armstrong et al. [34]

For each dementia type, the clinical criteria used are provided in chronological order. AD=Alzheimer's disease dementia, FTD=frontotemporal dementia, VaD=vascular dementia, DLB=Lewy Body disease, PSP=progressive supranuclear palsy, CBD=corticobasal degeneration.
