# Supplementary material for: High occurrence of transportation and logistics occupations among vascular dementia patients: an observational study
Source: Alzheimers Res Ther. 2019 Dec 27;11:112. doi: 10.1186/s13195-019-0570-4 (PMC6933928; doi:10.1186/s13195-019-0570-4)
Supplement: Supplementary file 3 — Additional file 3: Table S2. Distribution of dementia types across occupations groups after excluding comorbid cases. [file 13195_2019_570_MOESM3_ESM.pdf]

Table S2. Distribution of dementia types across occupations groups after excluding comorbid cases

	Total	AD	FTD	VaD	DLB	PSP/CBD
**Total (n, [%])**	2,028 (100)	1,401 (69)	274 (14)	83 (4)	171 (8)	99 (5)
**Pedagogical (n, [%])**	172 (100)	122 (71)	24 (14)	5 (3)	14 (8)	7 (4)
**Creative/Linguistic (n, [%])**	72 (100)	52 (72)	8 (11)	4 (6)	6 (8)	2 (3)
**Commercial (n, [%])**	195 (100)	138 (71)	28 (14)	5 (3)	17 (9)	7 (4)
**Business/Administrative (n, [%])**	374 (100)	265 (71)	46 (12)	15 (4)	27 (7)	21 (6)
**Management (n, [%])**	195 (100)	135 (69)	17 (9)	3 (2)	24 (12)*	16 (8)*
**Governmental/Law/Safety (n, [%])**	88 (100)	63 (72)	10 (11)	1 (1)	9 (10)	5 (6)
**Technical (n, [%])**	409 (100)	261 (64)*	69 (17)*	24 (6)*	39 (10)	16 (4)
**Agricultural (n, [%])**	30 (100)	15 (50)*	7 (23)	1 (3)	3 (10)	4 (13)*
**Health Care/Welfare (n, [%])**	271 (100)	211 (78)***	26 (10)*	7 (3)	13 (5)*	14 (5)
**Service (n, [%])**	135 (100)	94 (70)	23 (17)	8 (6)	7 (5)	3 (2)
**Transportation/Logistics (n, [%])**	87 (100)	45 (52)***	16 (18)	10 (11)***	12 (14)	4 (5)

10 cells (18.2%) had an expected count less than 5; the minimum expected count was 1.23. AD=Alzheimer's disease dementia, FTD=frontotemporal dementia, VaD=vascular dementia, DLB=Lewy Body disease, PSP=progressive supranuclear palsy, CBD=corticobasal degeneration. *Chi_2 adjusted residual is ≤ -2 or ≥2 (corresponding to p<.05), ***Chi_2 adjusted residual is ≤ -3 or ≥3 (corresponding to p<.001).
