# Supplementary material for: High occurrence of transportation and logistics occupations among vascular dementia patients: an observational study
Source: Alzheimers Res Ther. 2019 Dec 27;11:112. doi: 10.1186/s13195-019-0570-4 (PMC6933928; doi:10.1186/s13195-019-0570-4)
Supplement: Supplementary file 4 — Additional file 4: Table S3. Distribution of dementia types across occupations groups after excluding cases with multiple occupations. [file 13195_2019_570_MOESM4_ESM.pdf]

Table S3. Distribution of dementia types across occupations groups after excluding cases with multiple occupations

	Total	AD	FTD	VaD	DLB	PSP/CBD
**Total (n, [%])**	1,853 (100)	1,282 (69)	246 (13)	83 (5)	154 (8)	88 (5)
**Pedagogical (n, [%])**	155 (100)	110 (71)	21 (14)	5 (3)	11 (7)	8 (5)
**Creative/Linguistic (n, [%])**	62 (100)	45 (73)	8 (13)	2 (3)	5 (8)	2 (3)
**Commercial (n, [%])**	73 (100)	120 (69)	27 (16)	5 (3)	14 (8)	7 (4)
**Business/Administrative (n, [%])**	356 (100)	250 (70)	43 (12)	15 (4)	28 (8)	20 (6)
**Management (n, [%])**	199 (100)	139 (70)	17 (9)*	3 (2)*	24 (12)*	16 (8)*
**Governmental/Law/Safety (n, [%])**	83 (100)	59 (71)	9 (11)	2 (2)	9 (11)	4 (5)
**Technical (n, [%])**	360 (100)	233 (65)*	60 (17)*	25 (7)*	30 (8)	12 (3)
**Agricultural (n, [%])**	25 (100)	12 (48)*	6 (24)	1 (4)	3 (12)	3 (12)
**Health Care/Welfare (n, [%])**	245 (100)	193 (79)***	23 (9)	6 (2)	12 (5)*	11 (5)
**Service (n, [%])**	117 (100)	81 (69)	19 (16)	8 (7)	7 (6)	2 (2)
**Transportation/Logistics (n, [%])**	78 (100)	40 (51)***	13 (17)	11 (14)***	11 (14)	3 (4)

10 cells (18.2%) had an expected count less than 5; the minimum expected count was 1.12. AD=Alzheimer's disease dementia, FTD=frontotemporal dementia, VaD=vascular dementia, DLB=Lewy Body disease, PSP=progressive supranuclear palsy, CBD=corticobasal degeneration. *Chi2 adjusted residual ≤ -2 or ≥2 (corresponding to p<.05), ***Chi2 adjusted residual ≤ - 3 or ≥3 (corresponding to p<.001).
