# Supplementary material for: High occurrence of transportation and logistics occupations among vascular dementia patients: an observational study
Source: Alzheimers Res Ther. 2019 Dec 27;11:112. doi: 10.1186/s13195-019-0570-4 (PMC6933928; doi:10.1186/s13195-019-0570-4)
Supplement: Supplementary file 5 — Additional file 5: Table S4. Uncorrected and corrected logistic regression models of relationships between occupational class and dementia type. [file 13195_2019_570_MOESM5_ESM.pdf]

**Table S4. Uncorrected and corrected logistic regression models of relationships between occupational class and dementia type**

| | | Uncorrected | | | Corrected | | |
|---|---|---|---|---|---|---|---|
| **VaD** | **β** | **OR** | **P-value** | **β** | **OR** | **P-value** | **CIE (β)** |
| **Transportation/Logistics[a]** | 1.23 | 3.41 | .004 | 1.04 | 2.84 | .018 | 14.8 % |
| **AD** | **β** | **OR** | **P-value** | **β** | **OR** | **P-value** | **CIE (β)** |
| **Transportation/Logistics[b]** | -.85 | .43 | .001 | -.62 | .54 | .020 | 27.4 % |
| **Health Care/Welfare[b]** | .55 | 1.74 | .003 | .31 | 1.37 | .101 | 43.2 % |

VaD=vascular dementia, AD=Alzheimer's disease dementia, OR=odds ratio, CIE=change-in-estimate. We used a forward selection procedure with a change-in-estimate (CIE) criterion of 10% [42,43] to select relevant covariates among all participants with complete data (n=1,562/2,121). We tested age, sex, education and VRFs as covariates; only VRFs and sex were finally included.
