# Supplementary figures and images for: High occurrence of transportation and logistics occupations among vascular dementia patients: an observational study
Source: Alzheimers Res Ther. 2019 Dec 27;11:112. doi: 10.1186/s13195-019-0570-4 (PMC6933928; doi:10.1186/s13195-019-0570-4)

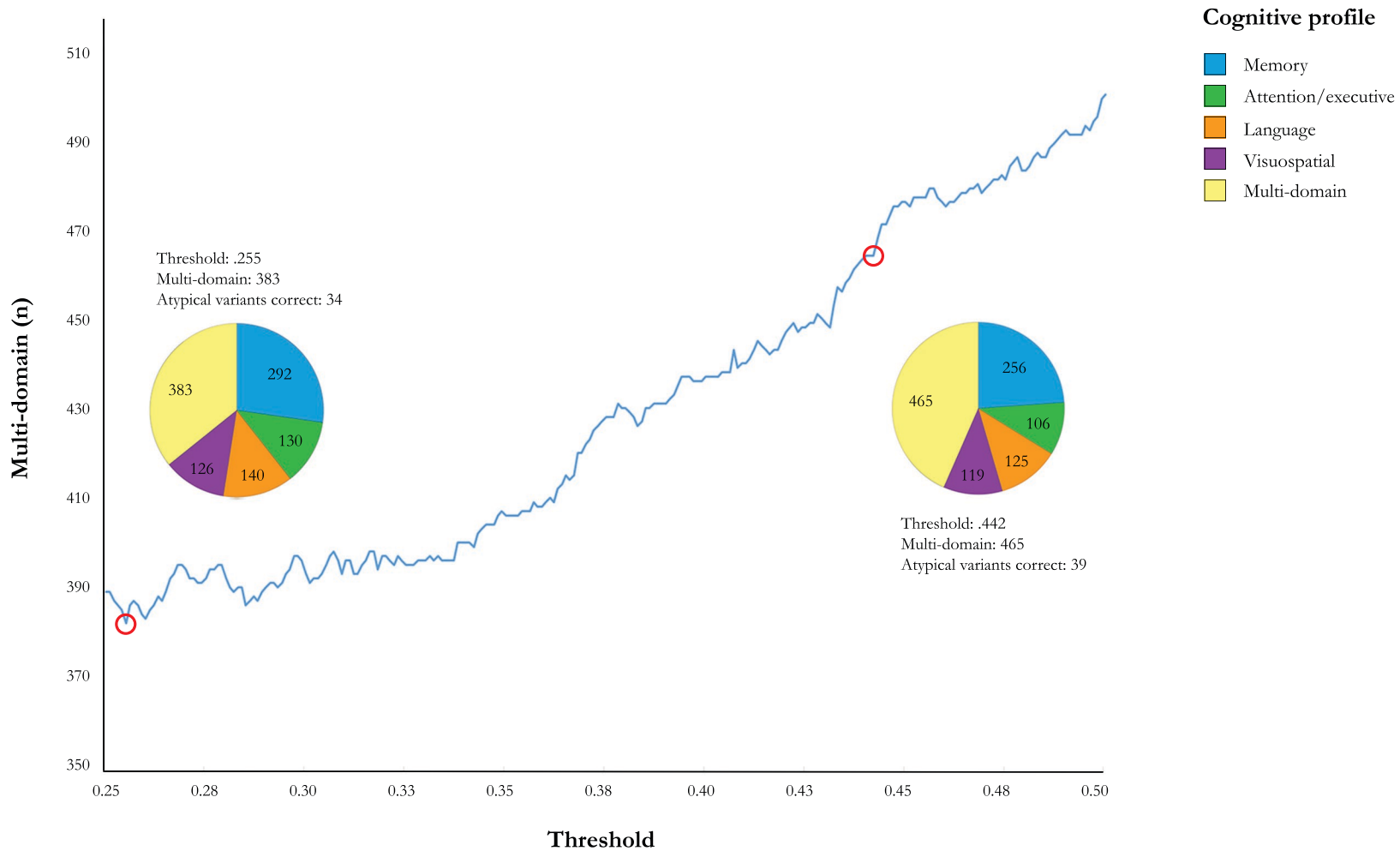

Supplement: Supplementary file 6 — Additional file 6: Figure S1. Thresholds used for the dichotomization of cognitive domains and division of participants across cognitive profiles. Within the AD subsample (n=1,071), we dichotomized each cognitive domain score (i.e. memory, attention/executive functions, language and visuospatial functions), based on whether or not a domain’s W-score was considerably lower compared to an individual’s global cognition score. We used an optimal threshold between .250 and .500 for this dichotomization, and defined “optimality” in two different ways: 1) the lowest number of participants in the multi-domain profile and the lowest sum of squared group sizes (threshold=.255), 2) the highest number of atypical variant AD cases (n=85) categorized into the language (i.e. for logopenic aphasia) or visuospatial cognitive profile (i.e. for PCA; n=.442). Note that neither thresholds (i.e. .255 or .442) resulted in all atypical variants being categorized into these two cognitive profiles, presumably because in advanced disease stages, several cognitive domains become affected. For many participants with an initial logopenic aphasia or PCA diagnosis, visuospatial/language may no longer have been predominant at study inclusion, causing them to be assigned to the multi-domain instead. [file 13195_2019_570_MOESM6_ESM.pdf]

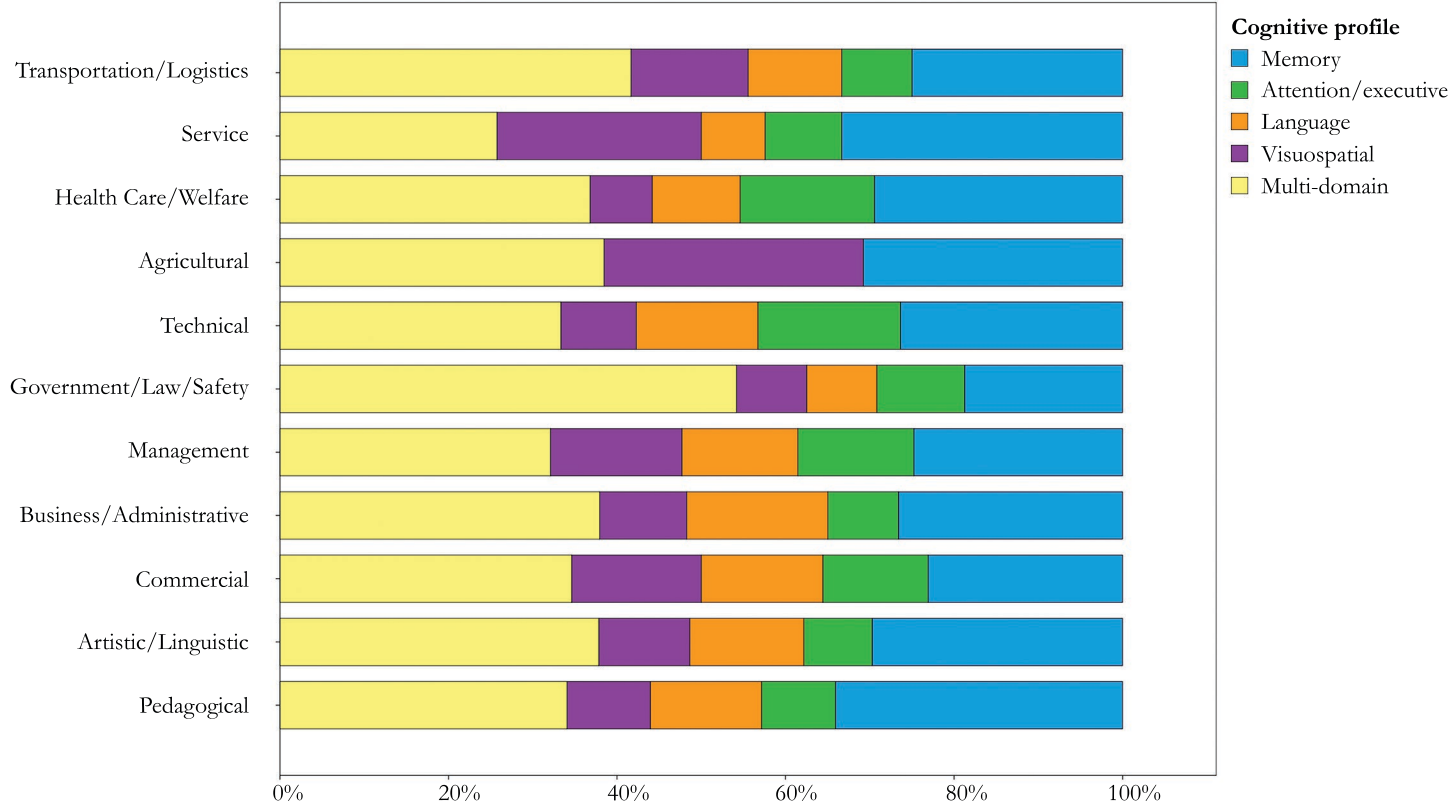

Supplement: Supplementary file 7 — Additional file 7: Figure S2. Proportions of AD-related cognitive profiles for each occupational class. There were no significant differences between groups (X2 n.s.). [file 13195_2019_570_MOESM7_ESM.pdf]

A)

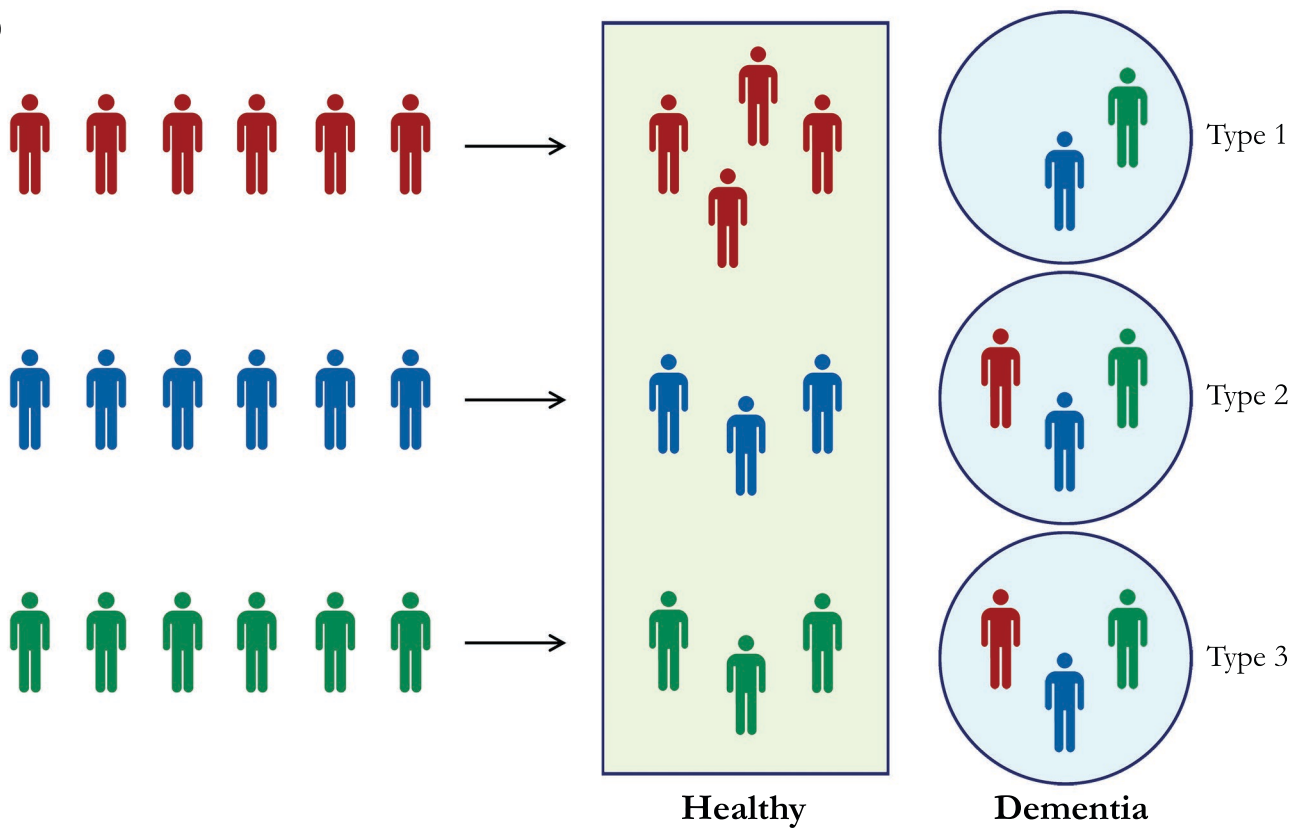

B)

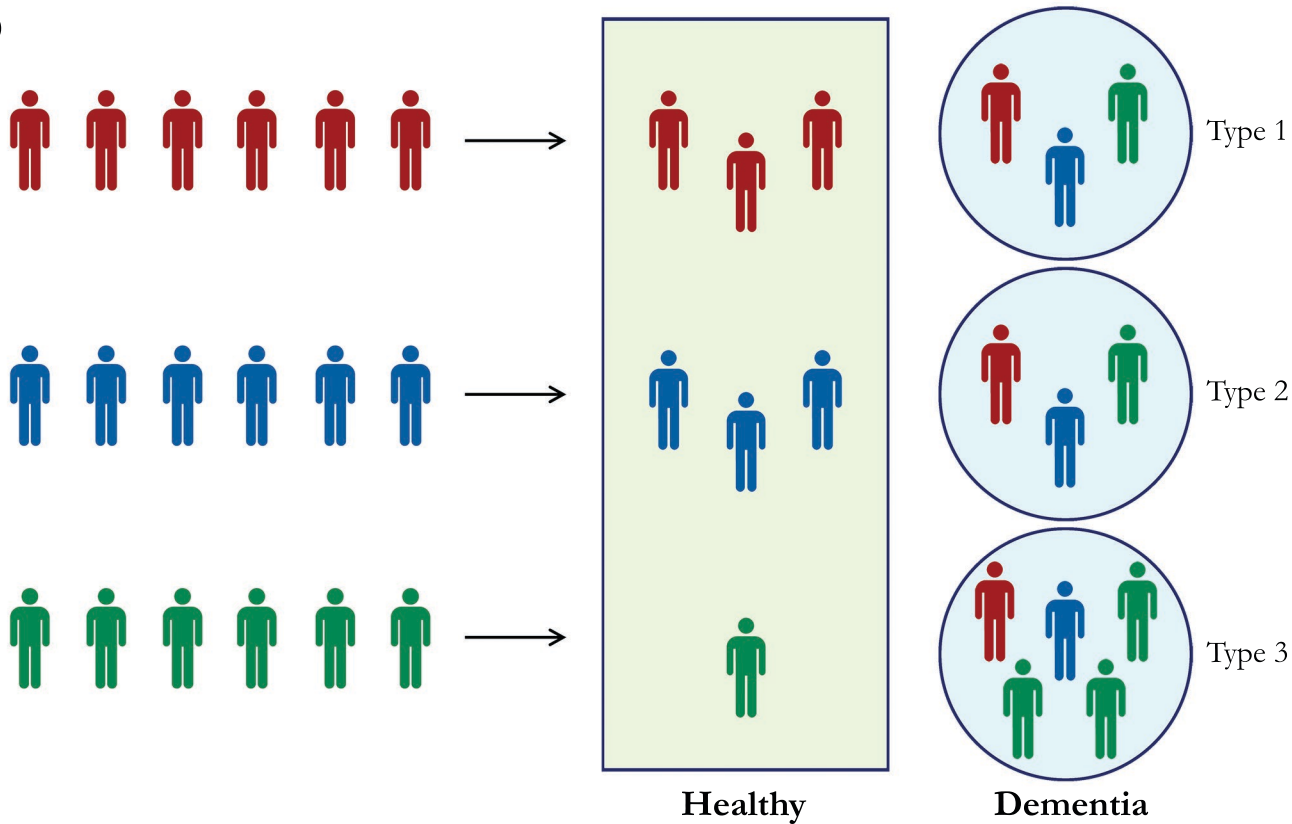

Supplement: Supplementary file 8 — Additional file 8: Figure S3. Schematic representation of protective and harmful effects and resulting cells in the contingency table. Each color (red, blue, green) represents a different occupational class. In scenario A, the red occupational class is relatively protected against dementia type 1, while the distribution of the remaining dementia types across occupations is equal. When the healthy control group is included in the analysis, this protective effect will be reflected in a higher proportion of individuals in red in the healthy group (4/6=67% versus 50% for blue/green) and a lower percentage of red persons in the type 1 dementia group (0% versus 1/6=17% for blue/green). All other proportions will be identical across occupational classes. However, when the healthy control group is not taken into account, the protective effect of the red occupation for dementia type 1 will create also an apparent “harmful” effect of the red occupation for dementia types 2 and 3 (i.e. 1/2=50%, compared to 1/3=33% for blue/green). In scenario B, the green occupational class shows a harmful effect on the development of dementia type 3, while no other differences between groups exist. Again, when the healthy control group is included, this only results in a lower percentage of healthy green individuals (1/6=17% versus 50% for red/blue) and a greater proportion of persons with green occupations in dementia type 3 (i.e. 50% versus 1/6=17% for red/blue). In the absence of a healthy control group, however, the green occupational class’ harmful effect for dementia type 3 leads to an apparent “protective” effect for dementia types 1 and 2 (1/5=20%, compared to 1/3=33% for red/blue). [file 13195_2019_570_MOESM8_ESM.pdf]
